# Combination of thoracic epidural analgesia with patient-controlled intravenous analgesia versus traditional thoracic epidural analgesia for postoperative analgesia and early recovery of laparotomy: a prospective single-centre, randomized controlled trial

**DOI:** 10.1186/s12871-022-01891-3

**Published:** 2022-11-07

**Authors:** Wenwen Xu, Youpei Li, Nanqi Li, Yu Sun, Chao Wang, Ke An

**Affiliations:** 1 Department of Anaesthesiology, the First Affiliated Hospital of Sun Yat-Sen University, Guangzhou, 510080 China; 2 Department of Anaesthesiology, Sun Yat-Sen Memorial Hospital, Sun Yat-Sen University, Guangzhou, 510080 China; 3 Department of Anaesthesiology, Sun Yat-Sen University Cancer Center, Guangzhou, 510080 China

**Keywords:** Epidural analgesia, Patient-controlled intravenous analgesia, Postoperative analgesia, Enhanced recovery, Laparotomy

## Abstract

**Background:**

Thoracic epidural analgesia (TEA) has always been the first choice for postoperative pain treatment, but associated complications and contraindications may limit its use. Our study put forward a new analgesic strategy that combines TEA with patient controlled intravenous analgesia (PCIA) to optimize TEA.

**Methods:**

Patients undergoing laparotomy were enrolled in this prospective randomized study. Patients were randomized to one of two groups: TEA/PCIA group and TEA group. Patients in TEA/PCIA group received TEA in the day of surgery and the first postoperative day and PCIA continued to use until the third postoperative day. Patients in TEA group received TEA for three days postoperatively. Visual analogue scale (VSA) pain scores at rest and on movement at 6, 24,48,72 h after surgery were recorded. In addition, the incidence of inadequate analgesia, adverse events, time to first mobilization, time to pass first flatus, time of oral intake recovery, time of urinary catheter removal, postoperative length of hospital stay, cumulative opioid consumption, and the overall cost were compared between the two groups. We examined VAS pain scores using repeated measures analysis of variance; *P* < *0.05* was considered as statistically significant.

**Results:**

Eighty-six patients were analysed (TEA/PCIA = 44, TEA = 42). The mean VAS pain scores at rest and on movement in TEA/PCIA group were lower than TEA group, with a significant difference on movement and 48 h postoperatively (*P* < 0.05). The time to first mobilization and pass first flatus were shorter in TEA/PCIA group (*P* < 0.05). Other measurement showed no statistically significant differences.

**Conclusions:**

The combination of TEA with PCIA for patients undergoing laparotomy, can enhance postoperative pain control and facilitate early recovery without increasing the incidence of adverse effects and overall cost of hospitalization.

**Trial registration:**

Chinese Clinical Trial Registry(www.chictr.org.cn), ChiCTR 1,800,020,308, 13 December 2018.

## Introduction

Enhanced recovery after surgery (ERAS) is a standardized and evidence-based perioperative care protocol and has been developed to many surgical fields. It largely facilitates postoperative recovery and attenuates peri-operative stress response and thus reduces complications and length of stay [1–3]. Adequate postoperative analgesia has always been considered as one of the key components for ERAS programs. Poor pain control would lead to delayed recovery and increased morbidity and bring challenges to subsequent treatment. Several analgesic techniques or drugs have been created and widely used for postoperative acute pain management within the past 20 years, however the outcomes of pain control are not always satisfactory. Correll et al. published a scientometric analysis pointed out that inappropriate use of new technologies and drugs would impede improvement on postoperative acute pain relief [4].

Thoracic epidural analgesia (TEA), as the cornerstone of postoperative pain relief in laparotomy, can provide better effective pain management compared with patient controlled intravenous analgesia (PCIA) [5]. Prior studies have supported that TEA could reduce the incidence of postoperative pulmonary complications and facilitate the recovery of gastrointestinal function [6, 7]. However, some problems still emerge in the application of TEA, such as postoperative hypotension, fluid overload, urinary retention, and motor block. Furthermore, a review summarizes that the failure of epidural anaesthesia and analgesia occurs in up to 30% in clinical practice [8]. Current guidelines for ERAS still emphasize the role of TEA in multimodal analgesia for postoperative pain control. Thus, how to optimize TEA is important to laparotomy.

**Fig. 1:**
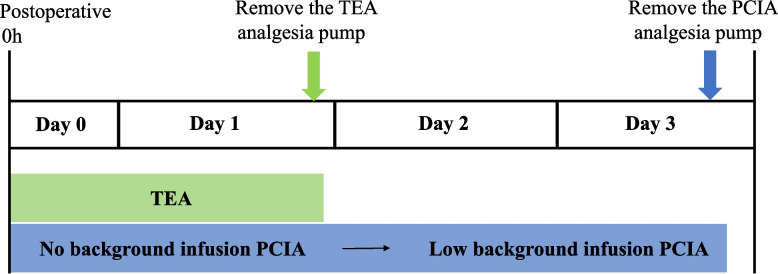
The protocol and flow diagram of the TEA/PCIA

To our knowledge, laparotomy is often characterized by severe trauma, severe pain, and long recovery time. PCIA is not recommended for laparotomy because of its low efficacy and a higher rate of adverse events. However, combination of different classes of analgesics in PCIA, along with the advantage of rapid onset, may improve efficacy or minimize adverse effects. Therefore, under the concept of multimodal analgesia, our study put forward a new analgesic strategy that combines short-term TEA with PCIA on the first two postoperative days and apply PCIA alone afterwards in the subsequent two days (Fig. 1). This strategy could not only maximize the effect of epidural analgesia, but also theoretically reduce the adverse effects [9, 10]. This study attempted to take a multimodal analgesic approach to optimize postoperative analgesia and facilitate enhanced recovery. It is expected that the combination of TEA and PCIA would result in decreased pain scores, but it is uncertain that this approach could reduce pain scores without increasing costs or adverse effects. Therefore, we conducted a prospective non-blinded randomised controlled trial to compare TEA/PCIA with TEA, to explore the feasibility of combination of TEA with PCIA in pain control and early recovery after laparotomy under the goal of ERAS.

## Methods

This study was a single-centre prospective non-blinded randomised controlled trial (Chinese Clinical Trial Registry, ChiCTR 1,800,020,308, 13/12/2018). Ethical approval for this study (Ethical Committee No. [2018]265) was provided by the Ethics Committee of the First Affiliated Hospital of Sun Yat-Sen University. The study adhered to the CONSORT guidelines.

### Participants

A total of 102 patients undergoing laparotomy in the First Affiliated Hospital of Sun Yat-Sen University were recruited between December 2018 and December 2019. The patients aged 18–75 years, with an ASA I or II, and BMI ranged from 18 to 27 kg m^−2^, who were undergoing laparotomy (hepatectomy, pancreaticoduodenectomy, gastrointestinal surgery, or colorectal surgery), were eligible for this study. Patients were randomly allocated to group TEA/PCIA or TEA according to a random number table by the Social Sciences software version 20.0 (SPSS Inc, Chicago, IL, USA). All participants must be able to understand the research protocol and signed informed consent. Exclusion criteria included contraindication to epidural analgesia, allergy or sensitivity to local anaesthetics, contraindication to opioid and non-opioid analgesic drugs. The patients with a history of chronic pain or long-time medication with antidepressants, narcotic analgesics or nonsteroidal anti-inflammatory drugs (NSAIDs) were also excluded.

Patients may discontinue participation in the trial at their own request, or be withdrawn if a surgery is not performed, or continuation of the trial may be detrimental to the patient’s health in the investigator’s opinion. Drop-out patients will be included in the final report to ensure complete transparency of the trial.

### Preparations in the operation room before surgery

After established intravenous access and continuous monitoring in the operative room, the patients were placed in the lateral position to receive TEA prior to the induction of general anaesthesia. Insertion of an epidural catheter was performed between T8 and T10 in patients undergoing a right sided colon resection or upper abdominal surgery (hepatectomy, pancreaticoduodenectomy, gastrointestinal open surgery), or between T10 and T11 in patients undergoing a left sided colon resection. After the epidural space was identified using the loss of resistance technique with air, standard aseptic insertion procedure was performed. A test dose of 3 mL of 2% lidocaine was injected to ensure the catheter was in the correct space. Sterile device was used to hold the catheter in place after excluding the spinal anaesthesia.

### Standard general anaesthesia

All patients in the trial underwent a general anaesthesia. Anaesthesia was induced with sufentanil (0.3–0.5 mcg kg^−1^), cisatracurium (0.2 mg kg^−1^) or rocuronium (0.6 mg kg^−1^), propofol (2–3 mg kg^−1^). Standard monitoring used in the surgery involved electrocardiogram, blood pressure, respiratory rate, oxygen saturation, end-tidal carbon dioxide, central venous pressure, temperature, and Narcotrend® (MonitorTechnik, Bad Bramstedt, Germany). Anaesthesia was maintained by propofol and sevoflurane, as the depth of anaesthesia showed as Narcotrend® value was kept between 40 and 60.

### Intervention in TEA/PCIA group

Half an hour before the completion of surgery, 0.4 mg of hydromorphone 2 mL and 5 mL of 0.25% ropivacaine were injected into the epidural space as a loading dose. All the patients were then connected with an epidural analgesia pump (Jiangsu REHN Medical Instruments Technology CO., ITD). As for analgesia regimen, 0.125% ropivacaine combined with hydromorphone was used for TEA, with a background infusion rate of 2 mL h^−1^. TEA was only applied in the day of surgery and the first postoperative day. Hydromorphone combined with flurbiprofen was used for PCIA until third postoperative day. The removal time of an analgesia pump was recorded, and the cumulative opioid consumption was recorded in equivalents of oral morphine equivalents (OMEs) [11]. The types of medications and additional analgesics were documented in detail.

### Intervention in TEA group

The patients in TEA group received epidural puncture and catheterization to establish epidural analgesia before anaesthesia induction. TEA was used until third postoperatively day. The analgesia regimen for TEA was the same as that in TEA/PCIA group, with the analgesia pump settings of a background infusion rate of 2 mL h^−1^. Similarly, detailed recording included removal time of an analgesia pump, cumulative opioid consumption, and additional analgesics.

### Date collection

The demographic and operation-related information including age, sex, BMI, ASA grade, comorbidities, surgical type, incision type, and operation time was collected. Postoperative pain at rest and on movement was evaluated with visual analogue scale (VAS) pain score. The primary endpoints were mean VAS pain scores at rest and on movement for three days postoperatively. The secondary endpoints included VAS pain scores at rest and on movement at 6, 24, 48 and 72 h postoperatively, incidence of inadequate analgesia, incidence of opioid-related adverse events, the time to first mobilization, the time to pass first flatus, the time of oral intake recovery, the time of the urinary catheter removal, postoperative length of hospital stay (PLOS), cumulative opioid consumption, and overall cost.

### Sample size

The mean VAS pain scores at rest and on movement for three days postoperatively were the primary endpoints in our work. Kelly et al.reported the minimum clinically significant VAS pain score in the management of severe pain was 1 cm [12]. Standard deviations (SD) varying between 1.4 and 1.8 cm have been reported, thus we estimated a SD of 1.5 cm for the study. To achieve 90% power to detect a difference (1 cm) in the primary endpoints with a two-sided 5% level of significance, a sample size of 38 patients in each group of the study is needed. An additional four participants were recruited in each study arm to cover a maximum of 10% losses, thus the sample size required for each group was up to 42 subjects.

### Statistical analysis

SPSS software version 20.0 (SPSS Inc, Chicago, IL, USA) was used for statistical analysis. All numerical variables were first examined for normality. Numerical variables were descripted as mean ± SD for data with a normal distribution, otherwise descripted as median and interquartile range (IQR). Independent two-sample *t*-test was used for the comparison of the normally distributed numerical variables. The non-normally distributed numerical variables were compared by Mann–Whitney *U* test. Frequency and percentage were used for statistical description of categorical variables, and chi-square test or Fischer’s exact test were used for the comparison of the unordered categorical variables depend on their expected counts. Kruskal Wallis *H* test was used for ordinal multiple categorical variables. In addition, repeated measured data was analysed using repeated-measures analysis of variance (ANOVA), such as the VAS scores of the two groups at different time points. Bonferroni correction was used to adjust for the increased alpha error in the multiple comparisons. Survival analysis assessed by the Kaplan–Meier method and Breslow test was used to analyse postoperative indicators. *P* values < 0.05 was considered statistically significant (the level of significance was bilateral).

## Results

**Fig. 2:**
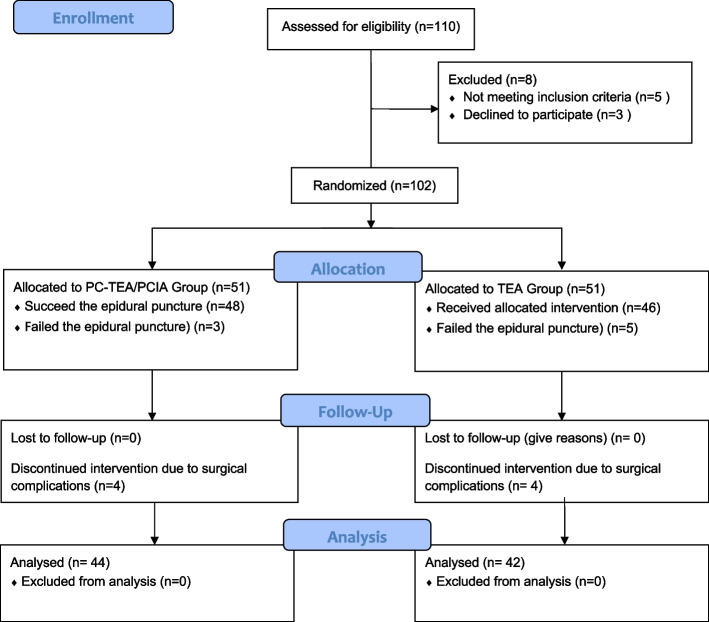
The CONSORT Flow Diagram In TEA/PCIA group, three patients failed to receive an epidural puncture, four patients withdraw from the research due to postoperative abdominal infection and haemorrhage. In TEA group, five patients failed to receive an epidural puncture, three were transferred to the ICU due to surgery complications, and one patient withdrew due to the changes in surgical protocols. TEA, thoracic epidural analgesia; PCIA, patient-controlled intravenous analgesia; ICU, intensive care unit

A total of 102 patients underwent laparotomy at our institution from November 2018 to November 2019 were recruited. Finally, 86 patients (44 patients in the TEA/PCIA group,42 patients in the TEA group) were included in the final statistical analysis, details of dropout reasons are given in Fig. 2. Baseline characteristics of the two groups are presented in Table 1(at the end of the manuscript). There was no significant difference in demographic characteristics, comorbidities, surgical type, and incision type between the two groups (*P* > 0.05). No significant differences were observed between the two groups in operation time, intraoperative fluid intake, intraoperative blood loss, intraoperative sufentanil consumption, cumulative opioid consumption, as well as length of stay and complications in the post anaesthesia care unit (PACU) (Table 2, at the end of the manuscript).

**Table 1:** Baseline characteristics of the included patients in the final statistical analysis

	TEA/PCIA Group	TEA Group	*P*
Age (y)	56.1 ± 11.1	54.6 ± 13.0	0.569
Sex (Male: Female)	36:8	33:7	0.705
BMI (kg m^−2^)	21.7 ± 2.8	22.2 ± 3.0	0.491
ASA I-II, n (%)	44	42	0.924
ASA I, n (%)	7 (16)	7 (17)	—
ASA II, n (%)	37 (84)	35 (83)	—
Comorbidities, n (%)	13 (29.5)	13 (31.0)	0.887
Diabetes, n (%)	7	4	—
Hypertension, n (%)	4	8	—
Respiratory, n (%)	2	4	—
Surgical type, n (%)	44	42	0.833
Liver surgery, n (%)	21 (47)	21 (50)	—
Pancreaticoduodenectomy, n (%)	10 (23)	12 (28)	—
Gastrointestinal surgery, n (%)	7(16)	5(12)	—
Colorectal surgery, n (%)	6 (14)	4 (10)	—
Incision type, n (%)	44	42	0.657
Reversed L-shaped incision, n (%)	1 (2.3)	1 (2.4)	—
Roof incision, n (%)	19 (43.2)	20 (47.6)	—
Subcostal incision, n (%)	2 (4.5)	2 (4.8)	—
Midline incision, n (%)	15 (34.1)	17 (40.4)	—
Para-midline incision, n (%)	7 (15.9)	2 (4.8)	—

Data are expressed as Mean ± SD, number (%). *TEA* thoracic epidural analgesia, *PCIA* patient-controlled intravenous analgesia, *BMI* body mass index, *ASA* American Society of Anaesthesiologists

**Table 2:** Operative characteristics, PACU variables and cumulative opioid consumption

	TEA/PCIA Group	TEA Group	*P*
Operation time (min)	267 [211 to 334]	247 [195 to 348]	0.694
Intraoperative sufentanil consumption (ug)	28.5 ± 6.2	29.1 ± 8.4	0.716
Intraoperative fluid intake (mL)	3250 [2700 to 3700]	3250 [2475 to 5025]	0.955
Intraoperative blood loss (mL)	300 [112.5 to 475]	225 [100 to5 25]	0.705
Blood transfusion, n (%)	12 (27.3)	12 (28.6)	0.893
PACU length of stay (min)	97.9 ± 40.4	104.0 ± 34.8	0.513
PACU complications, n (%)	6 (13.6)	8 (19)	0.952
Pain, n (%)	2 (4.5)	2 (4.8)	—
Dysphoria, n (%)	1 (2.3)	1 (2.4)	—
Shiver, n (%)	3 (6.8)	3 (7.1)	—
Hypertension, n (%)	0 (0)	1 (2.4)	—
Pain and dysphoria, n (%)	0 (0)	1 (2.4)	—
Cumulative opioid Consumption (mg)	41.48 [26.34 to 66.85]	38.64 [29.19 to 42.00]	0.109

Data are expressed as Mean ± SD, median [IQR], number (%). *TEA* thoracic epidural analgesia, *PCIA* patient-controlled intravenous analgesia, *PACU* post anaesthesia care unit

The mean VAS pain scores on movement during postoperative days 0–3 in TEA/PCIA group were significant lower (2.45 ± 0.55 vs 2.68 ± 0.52; *P* < 0.05) compared with TEA group (Table 3). TEA/PCIA group had lower VAS pain scores at rest and on movement at each time point compared with TEA group, with a significant difference at 48 h postoperatively (*P* < 0.05). In addition, inadequate analgesia occurred in 9 (20.5%) of the 44 patients in the TEA/PCIA group and in 13 (31%) of 42 patients in TEA group (*P* = 0.265), but it did not differ significantly between groups (Table 5).

**Table 3:** Mean VAS pain scores and VAS pain scores at various time points postoperatively

	TEA/PCIA Group	TEA Group	*P*
Mean R-VAS	1.18 ± 0.46	1.35 ± 0.50	0.093
Mean M-VAS	2.45 ± 0.55	2.68 ± 0.52	0.046
6-h R-VAS	1.64 ± 0.75	1.88 ± 0.80	0.15
6-h M-VAS	2.95 ± 0.86	3.12 ± 0.83	0.37
24-h R-VAS	1.36 ± 0.49	1.38 ± 0.70	0.89
24-h M-VAS	2.61 ± 0.62	2.79 ± 0.68	0.22
48-h R-VAS	0.95 ± 0.57	1.21 ± 0.52	0.03
48-h M-VAS	2.23 ± 0.64	2.64 ± 0.66	0.004
72-h R-VAS	0.75 ± 0.62	0.93 ± 0.75	0.23
72-h M-VAS	1.98 ± 0.59	2.19 ± 0.77	0.153

Data are expressed as Mean ± SD. *TEA* thoracic epidural analgesia, *PCIA* patient-controlled intravenous analgesia, *VAS* visual analogue scale, *R-VAS, VAS* score at rest, *M-VAS, VAS* score on movement

The TEA/PCIA group had earlier time to first mobilization and recovery of gastrointestinal motility (shorter time to first pass flatus) compared with the TEA group (*P* < 0.05). But no significant differences were observed in the postoperative length of hospital stay, the time to urinary catheter removal, and the time of oral intake recovery between the two groups (Table 4).

**Table 4:** Early postoperative recovery variables for the included patients

	TEA/PCIA Group	TEA Group	*P*
Time to first mobilization (d)	2 [2 to 3]	3 [2 to 4]	0.015
Time to first pass flatus (d)	2 [2 to 3]	3 [2 to 3]	0.048
Time of oral intake recovery (d)	4 [2 to 5]	3 [2 to 6]	0.513
Time of urinary catheter removal (d)	3 [2 to 4.75]	3 [2 to 4]	0.832
PLOS(d)	9 [7 to 11.75]	9.5 [8 to 13]	0.345

Data are expressed as median [IQR]. *TEA* epidural analgesia, *PCIA* patient-controlled intravenous analgesia, *PLOS* postoperative length of hospital stay

There was no significant difference in the incidence of opioid-related adverse events between the two groups (Table 5). The overall cost of hospitalization and the cost of anaesthesia were not different between the groups (Table 6).

**Table 5:** Incidences of inadequate analgesia and opioid-related adverse events

	TEA/PCIA Group	TEA Group	*P*
Inadequate analgesia	9 (20.5)	13 (31.0)	0.265
Nausea/vomiting, n (%)	9 (20.5)	8 (19.0)	0.269
Mild	3 (7.0)	3 (7.1)	0.269
Moderate	5 (11.4)	5 (11.9)	0.269
Severe	1 (2.1)	0 (0.0)	0.269
Hypotension, n (%)	11 (25.0)	8 (19.0)	0.506
Dizziness, n (%)	3 (6.8)	4 (9.5)	0.646
Pruritus, n (%)	0 (0.0)	2 (4.8)	0.143
Urinary retention, n (%)	1 (2.3)	2 (4.8)	0.529
Others*, n (%)	0 (0.0)	0 (0.0)	—

Data are expressed as number (%). *TEA* thoracic epidural analgesiam, *PCIA* patient-controlled intravenous analgesia; *: No respiratory depression, local anaesthetic intoxication, motor block, catheter prolapse were observed during the study. Patients with mean arterial pressure less than 65 mmHg were diagnosed with hypotension

**Table 6:** Overall cost and cost of anaesthesia^*^

	TEA/PCIA Group	TEA Group	*P*
Total cost (RMB)	75 011 [53 172 to 93 036]	75 773 [55 569 to 102 799]	0.777
Cost of anaesthesia (RMB)	5226 [4933 to 5740]	5171 [4658 to 5872]	0.412

Data are expressed as median [IQR]. *: A total of 82 patients were included in the statistical analysis, and four patients were excluded due to the special billing payment method in our hospital (three in TEA/PCIA group and one in TEA group). *TEA* thoracic epidural analgesia. *PCIA* patient-controlled intravenous analgesia

## Discussion

This study showed there was a significant reduction of mean M-VAS pain scores in TEA/PCIA group. Furthermore, there was significant lower VAS pain scores at 48 h postoperatively both in rest and on movement. In general, TEA/PCIA group provides superior pain control, which is consistent with our initial hypothesis. In past decades, epidural analgesia (EA) was regarded as the gold standard for treating postoperative pain after laparotomy. Previous publications reported that a successful epidural analgesia can provide excellent pain relief. But an epidural catheter was placed before surgery and used for 2–3 days postoperatively, many epidurals are not effective for such long periods [13]. Another literature review showed high analgesia failure rates of EA ranging from 13% to 48.6%, the main reasons reported were catheter dislodgment, malposition, occlusion and unplanned removal [14]. Some of these reasons is hard to detected and prevention. Once epidural failure, the patients need to tolerate the pain for a period, until additional drugs are used for relieving pain. In our study, the combination of short-term TEA with PCIA not only maximized analgesic effect, but also decreases the analgesia failure theoretically. Besides, opioids and NSAID were used for PCIA at the same time. Opioids can treat inadequate analgesia and reduce the regressed risk of sensory level of epidural analgesia, NSAID can effectively make up for the poor effect of TEA on inflammatory pain [15].

In our study, the time to first mobilization in TEA/PCIA group was earlier than that in TEA group, which may be attributed to better pain control. Additionally, early removal of epidural catheter may be another important factor. As we know, TEA has always been recognized for its analgesic effect, but some risks and complications, such as catheter dislodgment, increased risk of infection, adverse to early mobilization, adverse to the prevention of deep vein thrombosis, which could be limiting factors for ERAS programs [13]. Early removal of epidural catheter could decrease the incidence of motor block. At the same time, it also relieved the difficulty of postoperative care and labour-intensive monitoring. The patients will be more comfortable for mobilization without concerning the epidural catheter falling out.

Our results showed that the time to pass first flatus in TEA/PCIA patients was earlier than that in TEA patients. Previous studies have demonstrated that better pain control brings earlier mobilization, and thus contributes to recovery of gastrointestinal function and reduction of the risks of pulmonary and cardiovascular events [7, 16]. However, the result seems to be contradictory to the beneficial effect of TEA on the recovery of gastrointestinal function [6]. EA may promote a faster return in gastrointestinal motility via various mechanisms including decrease in opioid administration, blockade of the relevant sympathetic nerve and reduction of inflammatory reactions, but this advantage being increasingly doubted in recent reviews. The benefits of TEA in the recovery of gastrointestinal motility after colorectal surgery have been confirmed in several clinical trials, but for other types of laparotomies, its role on recovery of bowel function remains questionable. In a retrospective analysis of open surgery for gynecological tumors, EA was correlated with a higher incidence of ileus risk (odds ratio: 2.6; *P* = 0.03) [17]. In another prospective observational study on gastrointestinal function recovery after upper abdominal surgery, the first gas-out time was not different between the TEA group and PCIA group [18]. The study explained that the failure of TEA to promote gastrointestinal function recovery may attribute to the combination administration of local anesthetics and opioids in the epidural analgesia regimen. Moreover, the study also pointed out that the implementation of ERAS programs may also make TEA become less important on gastrointestinal function. Postoperative ileus is multifactorial and represents a limiting factor for the implementation of ERAS. The correlation between EA and postoperative ileus may require further research.

In the present study, the combination of TEA/PCIA required higher dosage of opioids compared with TEA alone. Therefore, whether the risk of opioid-related adverse events would increase was also the focus of this study. Our results showed that no significant difference was observed in the incidences of opioid-related adverse events between the two groups. The incidence of postoperative nausea and vomiting was less in both TEA/PCIA group (20.5%) and TEA group (19.0%), which is consistent with that incidence in our institution and lower than the generally reported incidence of 30–50% [19, 20]. Hypotension is an unwanted side effect of epidural analgesia. TEA/PCIA group did not show a lower incidence. Further analysis found that postoperative hypotension mainly occurred in the night of the first postoperative day. Postoperative hypotension of a major laparotomy is common and multifactorial. Due to the lack of a control group receiving PCIA alone, it is difficult to determine whether postoperative hypotension is attributable to epidural analgesia.

### Study limitations

There are several limitations in this study. The first is that this study was not blinded, as it was difficult to blind the observers and patients to the intervention they belonged. Secondly, given the lack of a PCIA control group, we cannot be sure if the adverse effects were attributed to the TEA itself. In addition, we utilized the scapula as a landmark rather than ultrasound guidance for the localization of an epidural catheter, and postoperative imagological examination was not routinely given to determine the location of the catheter. Therefore, we could not guarantee whether the catheter was located at the target place. Finally, the current study focused on patients undergoing major open surgery. Although the number of subjects included in the final analysis reached the requirement of sample size, the number of some subgroups was too small for further subgroup analysis. More attention will be paid to hepatobiliary surgery in our future clinical research.

## Conclusion

In summary, the combination of TEA and PCIA for patients underwent major open abdominal surgery, can provide superior postoperative analgesia and facilitate early rehabilitation without increasing the incidence rate of adverse effects and the overall cost of hospitalization.

## Data Availability

The full study protocol and raw data set can be obtained from the corresponding author (anke@mail.sysu.edu.cn).

## Abbreviations

TEA: Thoracic epidural analgesia
PCIA: Patient controlled intravenous analgesia
VAS: Visual analogue scale
R-VAS: VAS score at rest
M-VAS: VAS score on movement
ERAS: Enhanced recovery after surgery
ASA: American Society of Anesthesiology
BMI: Body mass index
NSAIDs: Nonsteroidal anti-inflammatory drugs
OMEs: Oral morphine equivalents
PLOS: Postoperative length of hospital stay
SD: Standard deviation
IQR: Interquartile range
PACU: Post anaesthesia care unit
ICU: Intensive care unit
EA: Epidural analgesia
